# Detection of Merkel Cell Polyomavirus DNA in Serum Samples of Healthy Blood Donors

**DOI:** 10.3389/fonc.2017.00294

**Published:** 2017-11-29

**Authors:** Elisa Mazzoni, John C. Rotondo, Luisa Marracino, Rita Selvatici, Ilaria Bononi, Elena Torreggiani, Antoine Touzé, Fernanda Martini, Mauro G. Tognon

**Affiliations:** ^1^Laboratories of Cell Biology and Molecular Genetics, Department of Morphology, Surgery and Experimental Medicine, Section of Pathology, Oncology and Experimental Biology, University of Ferrara, Ferrara, Italy; ^2^Department of Medical Sciences, Section of Microbiology and Medical Genetics, University of Ferrara, Ferrara, Italy; ^3^UMR INRA 1282 ISP, Faculté des Sciences Pharmaceutiques, Université Francois Rabelais, Tours, France

**Keywords:** Merkel cell polyomavirus, DNA, load, sequence, serum

## Abstract

Merkel cell polyomavirus (MCPyV) has been detected in 80% of Merkel cell carcinomas (MCC). In the host, the MCPyV reservoir remains elusive. MCPyV DNA sequences were revealed in blood donor buffy coats. In this study, MCPyV DNA sequences were investigated in the sera (*n* = 190) of healthy blood donors. Two MCPyV DNA sequences, coding for the viral oncoprotein large T antigen (LT), were investigated using polymerase chain reaction (PCR) methods and DNA sequencing. Circulating MCPyV sequences were detected in sera with a prevalence of 2.6% (5/190), at low-DNA viral load, which is in the range of 1–4 and 1–5 copies/μl by real-time PCR and droplet digital PCR, respectively. DNA sequencing carried out in the five MCPyV-positive samples indicated that the two MCPyV LT sequences which were analyzed belong to the MKL-1 strain. Circulating MCPyV LT sequences are present in blood donor sera. MCPyV-positive samples from blood donors could represent a potential vehicle for MCPyV infection in receivers, whereas an increase in viral load may occur with multiple blood transfusions. In certain patient conditions, such as immune-depression/suppression, additional disease or old age, transfusion of MCPyV-positive samples could be an additional risk factor for MCC onset.

## Introduction

DNA sequences of the Merkel cell polyomavirus (MCPyV) have been identified in Merkel cell carcinomas (MCC) (1). Subsequently, MCPyV sequences were detected in MCC in up to 80% of cases (2, 3), whereas Ig antibodies against MCPyV were revealed in approximately 80% of healthy subjects (3, 4) and MCC affected patients (3, 5). These results indicate that MCPyV, which is potentially oncogenic, is a ubiquitous human virus (3, 4).

Merkel cell carcinomas are a rare, aggressive skin cancer of neuroendocrine origin (3). Its incidence per year is estimated at about three/million, both in Europe and the United States (6). It should be noted that MCC has increased its frequency over the last two decades (3). MCC arises more frequently in elderly, white subjects of both genders, predominantly in sun-exposed areas, with an average onset age at presentation of 69 years old (7, 8). Its high-mortality rate may be partially explained by the advanced onset age, which results in the decreased immune function closely related to aging (9).

Immunosuppression has been demonstrated to be a risk factor for MCC development. Immunosuppressed individuals, such as those affected by AIDS, oncologic or hematological diseases, and organ transplant recipients, represent approximately 10% of MCC affected patients (3, 10). These MCC immunosuppressed patients have poorer prognoses (9). The role of immunosuppressive therapy in MCC pathogenesis is also suggested by reports of partial spontaneous regression of metastatic MCC after treatment discontinuation (11). It has been reported that MCC patients with high antibodies against MCPyV have a better clinical outcome and a reduced risk of recurrences (5). MCC does not always develop on the skin. Indeed, MCC onset may occur in other anatomical sites (8, 12).

The introduction of biologic drugs in treating some rheumatic diseases, such as rheumatoid arthritis and spondyloarthritis, while changing the outcome of these diseases, has shown some adverse effects including MCC onset (13). MCPyV is considered the main causal agent of MCC (1). MCPyV has been classified as a 2 A carcinogen by the WHO/IARC (3, 14). MCC onset, alongside MCPyV (15), needs other common risk factors, such as UV rays, aging, or immune system impairment (16). These risk factors can be seen in immunocompromised patients. MCPyV DNA has been identified in a wide variety of specimens, including blood donor buffy coats (3, 17).

Little attention has been given to the presence of MCPyV in the sera of healthy blood donors. In Transfusion Centers, leukodepletion is one of the methods employed to avoid the spread of unknown viral infections from blood donors to recipients (18). However, viruses or viral DNAs are potentially present in blood donor serum samples (19). These virus-positive samples could become the vector of viral infection in blood/hemoderivative receiver patients who may be affected by immune system impairment (13, 18, 19). In the present study, circulating MCPyV DNA coding sequences for the viral oncoprotein, large T antigen (LT), were investigated in serum samples from healthy blood donors using polymerase chain reaction (PCR) methods and DNA sequencing.

## Materials and Methods

### Serum Samples

Serum samples from healthy blood donors (*n* = 190) were collected from subjects in the 18–65 years old age range, with a median age of 45 years. Serum samples were collected at the Blood Bank of the University Hospital of Ferrara. Anonymously collected blood samples were coded with indications of age and gender only. Written informed consent from blood donors was obtained at the time of hospital admission. The County Ethics Committee, Ferrara, approved the project, assigning the number 151078 to this study, including the methods employed. All samples were stored at −80°C until DNA extraction.

### DNA Extraction, Qualitative, Quantitative (Q-PCR), and Droplet Digital PCR (ddPCR) Techniques

Total DNA was extracted from human sera (600 µl) using the commercial Charge Switch Forensic DNA Purification Kit (Thermo Fisher Scientific, Milan, Italy), following the manufacturer’s instructions. Purified DNA was eluted in 70 µl of buffer, whereas nucleic acid concentration and the presence of contaminants were assessed by OD reading with a NanoDrop 2000 (Thermo Scientific, Milan, Italy). DNA was stored at −80°C until time of analysis. To verify whether cross-contamination had occurred during the DNA extraction and PCR procedures, each sample was extracted and PCR amplified simultaneously with a salmon sperm DNA specimen and a mock sample lacking DNA (distilled water).

DNA was evaluated for its PCR suitability by amplifying the β-globin gene sequences (17).

**Figure 1:**
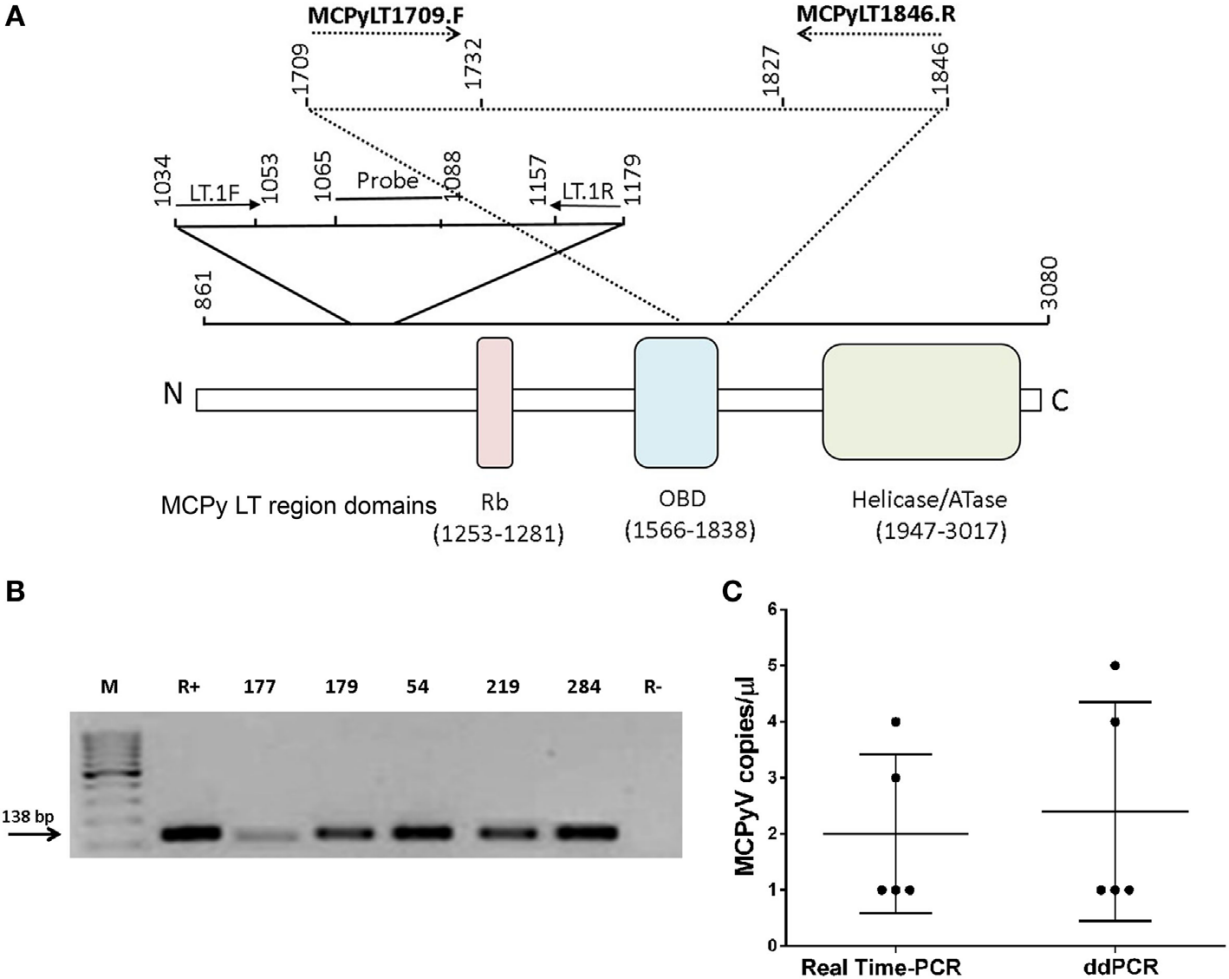
Merkel cell polyomavirus (MCPyV) DNA sequences in blood donor serum samples. **(A)** Schematic representation of the primers and probe employed in polymerase chain reaction (PCR), quantitative PCR, and droplet digital PCR (ddPCR) methods. **(B)** Agarose gel electrophoresis of PCR-amplified MCPyV large T antigen (LT) sequences, stained by ethidium bromide. M, molecular weight markers (100 bp). Lane R+, positive control represented by the recombinant plasmid pUC57MC1 carrying Merkel cell carcinomas 350 DNA sequences (17, 20). Lanes 1–5, DNA samples extracted from blood donor serum samples (number, *n*) (*n*177, *n*179, *n*54, *n*219, and *n*284). Lane R−, negative control of PCR reactions without DNA template. The arrow indicates the product size of 138 bp. **(C)** MCPyV copy number. MCPyV DNA load in blood donor serum samples is indicated as copy number per microliter of serum (left). Horizontal and vertical bars indicate the mean value and the SD, respectively.

Two different MCPyV LT coding sequences were investigated by qualitative PCR and real-time PCR (RT-PCR) analyses. In qualitative PCR, the primer sets employed were MCPyLT1709.F-MCPyLT1846.R (17), whereas in Q-PCR the primer sets LT.1F-LT.1R, and the LT probe were used, as previously described (17). In qualitative PCR, the recombinant plasmid pUC57MC1 (20) was introduced as positive control. This plasmid contains the MCC350 DNA, nt 2710-2899 (17, 20) (Table 1; Figure 1A).

**Table 1:** Primer sets employed by polymerase chain reaction (PCR) techniques to detect and quantify Merkel cell polyomavirus DNA sequences in serum samples from blood donors.

Primer sets	Nucleotide sequence position	Sequence 5'–3'	PCR amplicon (bp)
**PCR and ddPCR**			
MCPyLT1709.F	1,709–1,732	CAGGCATGCCTGTGAATTAGGATG	138
MCPyLT1846.R	1,846–1,827	TCAGGCATCTTATTCACTCC	
**Q-PCR**			
LT.1F	1,034–1,053	CCACAGCCAGAGCTCTTCCT	146
LT.1R	1,179–1,157	TGGTGGTCTCCTCTCTGCTACTG	
LT. probe	1,065–1,088	FAM-TCCTTCTCAGCGTCCCAGGCTTCA-TAMRA	

Specific quantitative Q-PCR assays, using TaqMan chemistry, were performed using the CFX96 Touch™ RT-PCR Detection System (Bio-Rad, Segrate, Milan, Italy) for MCPyV DNA load quantification. Recombinant plasmid pMCPyVLT.1, which contains 258 bp of the LT (21) coding sequence (FJ472933), was used as positive control. This recombinant plasmid contains viral DNA sequences of MCPyV strain named MCC 350, which is the main genotype circulating in the United States (21). Standard calibration curves were generated using 10-fold dilutions, from 10^1^ to 10^7^ copies, of pMCPyVLT.1 as reported previously (17). Cellular RNase P gene was used to determine the human cell equivalents of each sample under analysis (17). Samples and controls were analyzed three times by three different operators (Elisa Mazzoni, John C. Rotondo, and Rita Selvatici) in three independent experiments. Method sensitivity was 10 viral copies. Viral DNA loads are reported as MCPyV DNA copy per microliter. In addition, a new ddPCR method was developed for quantitatively determining the viral DNA load.

In our experiments, this ddPCR method does not need an internal-positive control because it detects, in absolute manner, viral DNA sequences. ddPCR was carried out by using MCPyLT1709.F-MCPyLT1846.R primer sets, which target LT coding sequences, giving a 138 bp product (Table 1). The ddPCR reaction mixture consisted of 11 µl of a 2× ddPCR super mix (QX200 EvaGreen ddPCR, Bio-Rad), 0.4 µl of each primer (MCPyLT1709.F, MCPyLT1846.R with a final concentration 0.181 µM for each one), and 10.2 µl of sample nucleic acid extracted from blood donors. MCPyV DNA load was determined as viral copies per microliter of serum sample (copies per microliter).

### DNA Sequencing

Amplicons from serum samples found to be MCPyV LT-positive were DNA sequenced (13) in order to verify MCPyV LT region specificity. MCPyV DNA sequencing data were compared with reference sequences from the National Center for Biotechnology Information (NCBI) Entrez Nucleotide database using the NCBI Blast program. To verify the presence of MCPyV DNA sequences, DNA from serum samples were analyzed by qualitative PCR with MCPyLT1709.F and MCPyLT1846.R primer sets, which generate an amplicon of 138 bp (17). The forward and reverse primers are located at nucleotide position 1,709–1,846, based on GenBank sequence EU375803. pUC57MC1 recombinant plasmid (20) carrying the MCC 350 strain DNA (1) was used as a positive control. Amplified MCPyV PCR products were purified using the QIAquick PCR Purification Kit (Qiagen, Milan, Italy) according to the manufacturer’s instructions. The MCPyV genotype was identified by direct sequence analysis. PCR amplicons were sequenced with the automated ABI Prism 3,730 × l Genetic Analyser (Applied Biosystems, Monza, Italy). The resulting MCPyV DNA sequences were BLAST vs. MCPyV DNA from different viral strains present in the NCBI database (http://www.ncbi.nlm.nih.gov/blast/Blast.cgi). Specifically, MCPyV DNA sequences were aligned against reference sequences from North America (MCC350, EU375803.1; MCC339, EU375804.1), Japan (TKS, FJ 464337), Sweden, France, and Italy MCPyV isolates (MKL-1, FJ173815) (22, 23). All experiments were carried out in laboratories with the standard biosecurity and safety procedures, according to institutional rules.

### Statistical Analysis

Merkel cell polyomavirus prevalence differences in positive serum samples from cohorts of healthy blood donors were determined using the χ^2^ test. All computational analyses were performed using Prism 6.0 (GraphPad software, San Diego, CA, USA). *P*-value was considered to be statistically significant when <0.05.

## Results

### MCPyV LT Sequence Detection in Blood Donor Serum Samples

In this study, DNA extracted from blood donor serum samples (*n* = 190) was analyzed by qualitative PCR for a small region of MCPyV LT sequences which encodes for the viral oncoprotein. This LT fragment of 138 bp, nt 1,709–1,846, maps in part at the 5' in the origin binding domain (OBD) and at the 3' end, outside the OBD region (Figure 1A). These MCPyV sequences were detected in 5 out of 190 (2.6%) of analyzed sera (Figure 1B). Interestingly, the five MCPyV-positive samples were from the cohort of older donors (46–65 years old). The prevalence of MCPyV DNA sequences detected in the cohort of subjects 18–45 years old differs from that of the cohort of healthy individuals 46–65 years old, but it was not statistically significant; this result is due to the absence of MCPyV DNA-positive samples in the cohort of younger individuals (0 vs. 5.3%; *P* > 0.05; Table 2).

**Table 2:** Merkel cell polyomavirus (MCPyV) DNA sequences identified by qualitative and quantitative polymerase chain reaction in serum samples of healthy blood donors.

Cohort year	Number of serum samples	MCPyV tag-positive sample/sample analyzed (%)
18–45	96	0/96 (0%)
46–65	94	5/94 (5.3%)
18–65	190	5/190 (2.6%)

*Human sera were from healthy blood donors. Statistical analysis was performed using the χ^2^ test. The prevalence of MCPyV DNA sequences detected in the cohort of subjects 18–45 years old differs from that of the cohort of healthy individuals 46–65 years old, but it was not statistically significant; this result is due to the absence of MCPyV DNA-positive samples in the cohort of younger individuals (0 vs. 5.3%; *P* > 0.05)*.

Then, the same 190 serum samples were analyzed further by Q-PCR for another LT sequence, the MCV T antigen unique region. The Q-PCR analysis was addressed to a segment of 146 bp, nt 1,034–1,179 (Table 1; Figure 1A). Results obtained by Q-PCR confirmed that 5/190 serum samples carry MCPyV LT sequences. The five MCPyV LT-positive samples, which were identified by Q-PCR matched the qualitative PCR analysis. Q-PCR revealed that in the five positive samples, MCPyV LT sequences were in the range of 1–4 copies/μl (mean value = 2 copies/μl), as demonstrated by the cycle threshold (ct) values (mean ct = 45) (Figure 1C). Our viral DNA loads obtained by Q-PCR are in agreement with previous studies, which reported a low-viral DNA load detected in other human specimens, such as nasopharyngeal aspirate samples (21), blood donor buffy coats (17), and serum samples, as well as in other tissues, from patients affected by inflammatory diseases (24). However, in other studies a high-viral load was detected in normal tissues, such as different skin anatomical sites (25).

As a control, and to confirm Q-PCR data, a ddPCR analysis was employed, using the same primer set of the qualitative PCR. This ddPCR technique is considered to be a precise, sensitive, and stable analytical method for overcoming problems relating to potential discrepancies in PCR analyses. Indeed, an absolute copy number of target DNA sequences can be detected directly by ddPCR, thus avoiding the use of positive controls and standard curve formulation. In the five MCPyV LT-positive samples, ddPCR revealed a viral DNA load in the range of 1–5 copies/μl (mean value = 2.4 copies/μl) (Figure 1C). Different PCR analyses carried out by investigating two distinct segments of MCPy LT sequences confirmed that five serum samples from healthy blood donors were MCPyV-positive, without discrepancy for samples found to be MCPyV-positive and viral DNA loads.

### Sequencing of MCPyV LT Sequences in Blood Donor Serum Samples

**Figure 2:**
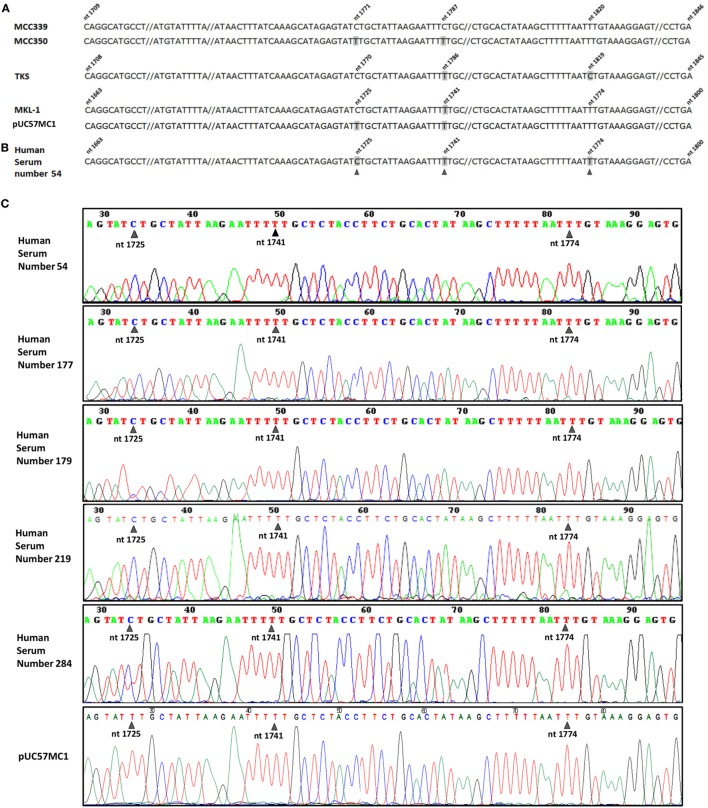
Merkel cell polyomavirus (MCPyV) sequence analysis. **(A)** MCPyV large T antigen (LT) sequences alignment of four MCPyV genotypes, Merkel cell carcinomas (MCC)339 (GenBank, accession number EU375804.1), MCC350 (GenBank, accession number EU375803.1), TKS (GenBank, accession number FJ464337), MKL-1 (GenBank, accession number FJ173815), together with the recombinant plasmid pUC57MC1, which contains MCC350 LT sequences (17, 20). The four aligned MCPyV DNA sequences, representing the same LT region, show different nucleotide numeration due to upstream nucleotide deletion (not shown) in their genome. Nucleotide substitutions in MCPyV strains are numbered and marked in gray. **(B)** DNA sequences of the MCPyV-positive sample, number 54. Five out of 190 DNA samples from serum samples contain single nucleotide substitutions (black arrow heads), which are cumulatively marked in gray in the representative MCPyV DNA sequences of sample 54. **(C)** MCPyV DNA sequences detected in human serum samples, numbers 54, 177, 179, 219, 284, contain a single nucleotide substitution at nt 1,725 (black arrow heads), corresponding to MKL-1 genotype, as shown in **(A)**, fourth line.

The five PCR amplicons in the MCPyV LT OBD/3' end domain were subjected to DNA sequencing. DNA sequence analysis enabled MCPyV LT sequences from the five MCPyV-positive serum samples to be identified and compared. This DNA analysis indicated that the viral sequences belong to MCPyV, whereas a comparative analysis between different MCPyV strains circulating in different populations suggested that the isolated samples used in this study are identical to the MCPyV circulating in Europe (Figure 2A). Indeed, the five MCPyV LT-positive samples carry viral DNA sequences which are undistinguishable from the MKL-1 strain, identified in Sweden, France, and Italy (17, 22) (Figures 2B,C). In this MCPyV nucleotide sequence of 97 nt, one single-nucleotide polymorphism (SNP) is considered sufficient to genotype four main different MCPyV strains, i.e., MKL-1, 350, 339, and TKS. Indeed, the five MCPyV DNAs analyzed are identical to MKL-1 strain. The other MCPyV strains differ each other for 1 nt. In the MCPyV 350 strain, at position 1,725, there is a transition C > T. In the MCPyV 339 strain, at position 1,741, there is another transition T > C. In the MCPyV TKS strain, at position 1,774, there is once again a transition T > C. These SNPs were always detected in the different MCPyV strains (13). This result is in agreement with other epidemiologic data obtained with a larger sample size (21, 22). Our DNA sequence analysis of the recombinant plasmid pUC57MC1, carrying MCC350 LT sequences, confirms the presence at position 1,725 of a T (Figure 2C), whereas in the GeneBank EU375803.1 this SNP, in the MCC350, is not reported.

## Discussion

This investigation was addressed to two viral LT gene sequences, which encode the MCPyV LT oncoprotein (1, 26). MCPyV LT DNA sequences were detected in sera from blood donors with a low prevalence and a low-viral DNA load. It should be noted that MCPyV DNA load is usually detected at low-copy number in different human tissues/samples, both from normal individuals and MCC patients. However, high-copy number was also reported (24). In a recent study, we reported a 0.06–1.2 viral DNA copy/cell range in three MCC specimens (13). This viral load is considered sufficient to contribute to MCC onset (27).

Since the first description of MCPyV in MCC (1), many studies have tried to identify the mechanism of DNA integration and tumor activation. In most MCC, MCPyV DNA integration into the host genome is associated with a particular molecular signature (1), i.e., the coding sequence for the *C*-terminal helicase domain of LT is mutated and/or deleted, while the *N*-terminal site is conserved. However, episomal MCPyV DNA could constitute a chronic antigenic stimulation, leading to the expansion of a lymphocytic clone, as proposed before for episomal MCPyV-positive skin samples (28).

Merkel cell polyomavirus LT sequences were detected in blood donor serum samples suggesting that this viral agent could be present in some blood leukocytes. In a recent investigation, MCPyV sequences were identified in blood samples from 44/8,000 (0.55%) individuals without PCR amplification (29). After primary infection, which occurs very early in life, MCPyV seems to remain in a latent/persistent state lifelong in immune competent hosts (4). The cellular protein SCF E3 ligase, targeting MCPyV LT oncoprotein seems to allow this small DNA tumor virus to remain in the latency state (30). Our data indicate that MCPyV may persist/reactivate in the host, whereas it can be detected at low prevalence and at low-DNA viral copy in the sera of immunocompetent healthy blood donors.

DNA sequence analysis of MCPyV LT DNA from the five positive serum samples showed high homology with MCPyV sequences belonging to the MKL-1 strain, which is the main MCPyV strain circulating in European Countries, such as France, Sweden, and Italy.

Since the MCPyV DNA used as control belongs to the American MCC 350 strain, our molecular data do not result from PCR contaminations or other technical artifacts. Indeed, the SNP C at nt 1,725 of MCPyV MKL-1 is present in the five serum samples found to be MCPyV-positive, whereas MCPyV MCC 350 sequences present in the recombinant plasmid pUC57MC1 carry a different SNP, i.e., a T, in the same nucleotide position. Thus, our data indicate that DNA contamination did not occur during PCRs from the recombinant plasmid. In addition, no contamination occurred by ddPCR because this method does not need an internal-positive control. ddPCR was used to confirm the viral DNA load, whereas DNA sequencing by the Sanger method assessed that PCR amplicons belong to the MCPyV MKL-1 strain (13).

It has been reported that about 10% of MCC cases are detected in immunosuppressed individuals (31), whereas it is well known that patients with impaired immune functions are more at risk of developing virus-related diseases (32). for instance, immunosuppression induced by HIV/AIDS in HIV-positive individuals increases the risk of developing MCC (10) compared with HIV-negative individuals (33). It is well established that the immune system counteracts MCPyV infection and associated MCC development. MCC patients with higher antibody titers show a better clinical outcome and a reduced risk of MCC recurrence (5). MCC regression occurs when immune suppression therapy is suspended/withdrawn (34). It is also known that pharmacological therapies with immunosuppressive agents used to prevent organ rejection result in immunodeficiency inducing skin cancer (35), non-melanoma skin cancer (36), and MCC (5). Previous reports (25) indicated a positive correlation between MCPyV virion-specific antibody titers and viral load at all anatomical sites tested (dorsal portion of the hands, forehead, and buttocks) (27). The mechanism through which productive MCPyV infections apparently persist despite robust anti-MCPyV antibody responses is unknown.

In this context, it should be recalled that many patients affected by different pathologies are dependent on blood transfusion from the time of diagnosis. These patients are exposed more than others to the risk of acquiring unknown/non-tested viral infections from blood donors. While the risk of blood transmissible viral infections for screened viruses continues to decline, new viruses such as MCPyV are becoming a concern. A recent investigation reported on a patient that developed MCC arising in an inguinal lymph node. This patient affected by von Willebrand disease received multiple blood transfusions (12).

We may speculate that elderly patients with immune system impairments could be high-risk receivers of MCPyV-positive blood samples.

## Ethics Statement

This study was carried out in accordance with the recommendations of University/Hospital of Ferrara guidelines, Ethics Committee of Ferrara, with written informed consent from all subjects. All subjects gave written informed consent in accordance with the Declaration of Helsinki. The protocol was approved by the Ethics Committee of Ferrara.

## Author Contributions

EM, AT, FM, and MT conceived and designed the experiments. LM, IB, and ET performed the PCR experiments. RS performed the sequencing experiments. JR performed the statistical analysis. EM and JR analyzed the data. EM and MT wrote the paper.

## Conflict of Interest Statement

The authors declare that the research was conducted in the absence of any commercial or financial relationships that could be construed as a potential conflict of interest.
